# AI-driven lightweight real-time SDR sensing system for anomalous respiration identification using ensemble learning

**DOI:** 10.1007/s42486-022-00113-6

**Published:** 2022-09-29

**Authors:** Umer Saeed, Qammer H. Abbasi, Syed Aziz Shah

**Affiliations:** 1 Research Centre for Intelligent Healthcare, Coventry University, Coventry, CV1 5FB UK; 2 James Watt School of Engineering, University of Glasgow, Glasgow, G12 8QQ UK; 3 School of Computing, Edinburgh Napier University, Edinburgh, EH10 5DT UK

**Keywords:** RF sensing, Ensemble learning, Software-defined radio, Anomalous respiration, Smart healthcare

## Abstract

In less than three years, more than six million fatalities have been reported worldwide due to the coronavirus pandemic. COVID-19 has been contained within a broad range due to restrictions and effective vaccinations. However, there is a greater risk of pandemics in the future, which can cause similar circumstances as the coronavirus. One of the most serious symptoms of coronavirus is rapid respiration decline that can lead to mortality in a short period. This situation, along with other respiratory conditions such as asthma and pneumonia, can be fatal. Such a condition requires a reliable, intelligent, and secure system that is not only contactless but also lightweight to be executed in real-time. Wireless sensing technology is the ultimate solution for modern healthcare systems as it eliminates close interactions with infected individuals. In this paper, a lightweight real-time solution for anomalous respiration identification is provided using the radio-frequency sensing device USRP and the ensemble learning approach extra-trees. A wireless software-defined radio platform is used to acquire human respiration data based on the change in the channel state information. To improve the performance of the trained models, the respiration data is utilised to produce large simulated data sets using the curve fitting technique. The final data set consists of eight distinct types of respiration: eupnea, bradypnea, tachypnea, sighing, biot, Cheyne-stokes, Kussmaul, and central sleep apnea. The ensemble learning approach: extra-trees are trained, validated, and tested. The results showed that the proposed platform is lightweight and highly accurate in identifying several respirations in a static setting.

## Introduction

The study of future pandemics and their relation to the coronavirus is currently an important topic of research. A coronavirus is a diverse group of viruses that comprise the Severe Acute Respiratory Syndrome (SARS-CoV), the Middle East Respiratory Syndrome (MERS-CoV) and the most recent virus, SARS-CoV-2, also known as COVID-19 (Singhal 2020). Whether coronavirus or any other disease that causes anomalous respiration (or shortness of breath) can cause mortality in a limited time (Li and Ma 2020). When an individual rests, respiration is commonly described as the number of breaths taken each minute. A typical respiration rate for humans is 10–24 breaths per minute. Anomaly (abnormal or irregular) respiration can be classified as hyperventilation (more than 24 breaths per minute) or hypoventilation (less than 10 breaths per minute). Other medical conditions, such as asthma, also affect the regular respiration rate. Therefore, the identification of anomalous respiration in real-time is of utmost importance (Yang and Yang 2020).

It is essential to accurately and promptly evaluate the respiration rate of infected individuals, as anomalous respiration measures can indicate a worsening of health. Identification of anomalous respiration normally requires the knowledge of healthcare personnel and is usually done in healthcare centres (Zhe et al. 2020). Spirometry which estimates airflow during the inhalation and exhalation processes is generally used in hospitals and clinics. Some of the other approaches include capnography, inductance pneumography, and electrical impedance pneumography (Liu et al. 2019). These approaches, however, necessitate hospitalisation and, due to the obvious medical emergency caused by a coronavirus, respiration monitoring of infected patients raises the danger of viral transmission among visitors to the hospitals (Siegel et al. 2021).

Most patients do not reveal major signs of respiration problems at the beginning, and therefore, healthcare personnel discharge them in order to self-monitor (Whiteside et al. 2020). However, as per medical research, individuals with minor medical conditions can also experience anomalous respiration, especially in the second or third week of being infected with coronavirus (Rehman et al. 2021a). Consequently, patients with normal respiration do not require hospitalisation and must be observed during self-isolation using a telemedicine approach (Huang et al. 2020). On the contrary, a real-time anomalous respiration identification system is needed for patients experiencing severe respiration problems (Rehman et al. 2021b).

**Fig. 1:**
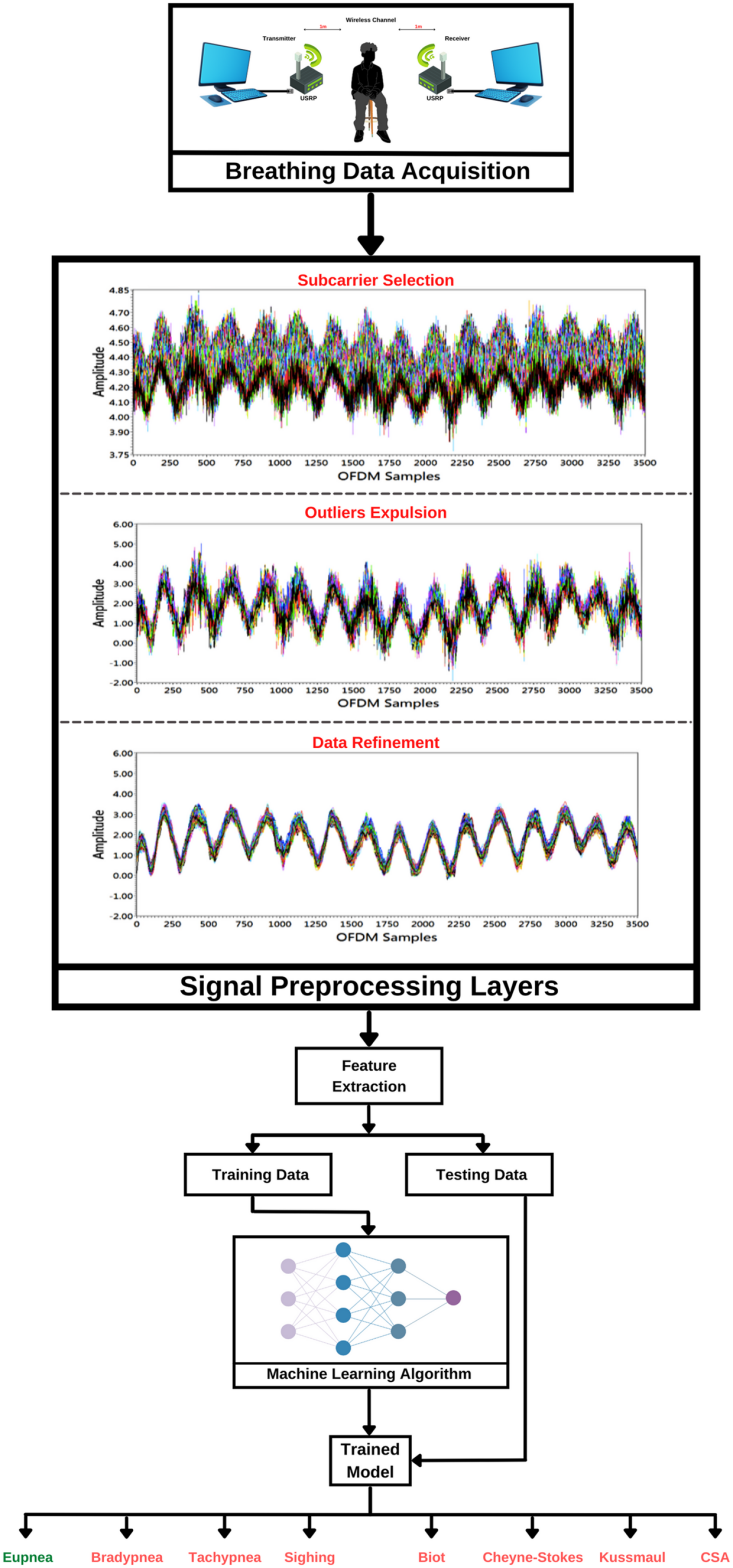
The proposed system framework for anomalous respiration identification

**Fig. 2:**
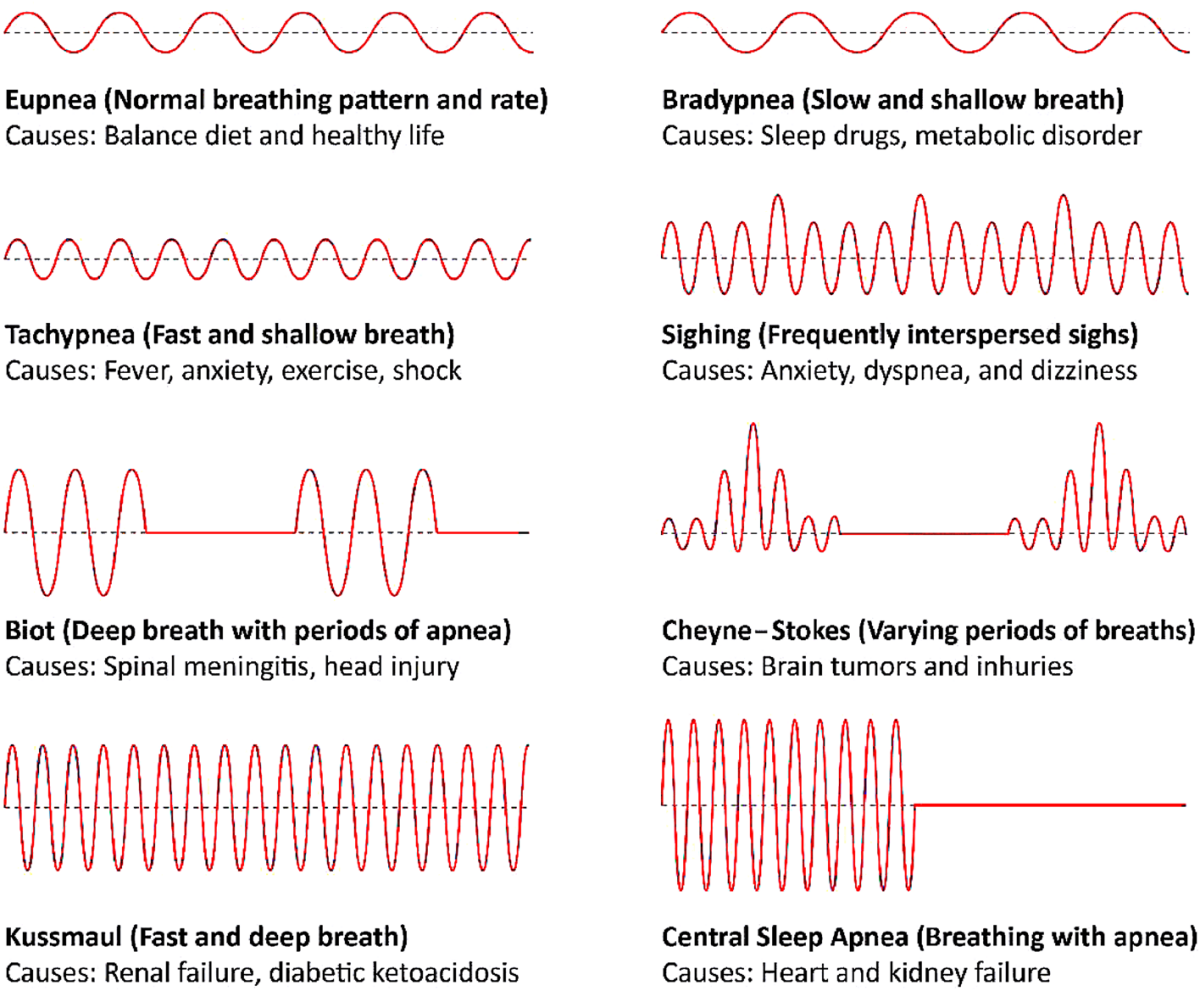
Diverse respiration patterns and its causes

In this paper, we proposed a lightweight real-time wireless system based on software-defined radio (SDR) and machine learning (ML) that together can be effectively used to identify various anomalous respiration. In the past, SDR and ML has been successfully used in several applications for the monitoring purposes (Saeed et al. 2021a; Usman et al. 2022; Saeed et al. 2021b). The framework of the proposed system is shown in Fig. 1. A breathing problem can cause a slow, shallow, fast, or deep breathing process. Based on variations in the data points, respiration patterns are classified into eight main types: eupnea, bradypnea, tachypnea, sighing, biot, Cheyne-stokes, Kussmaul, and central sleep apnea (CSA) (Whited and Graham 2017). Figure 2 shows different types of respiration patterns and their causes. There are several types of respiration patterns if focused on minor differences in their patterns. Nevertheless, this study is focused on the eight most common types of respiration patterns as illustrated in Fig. 2.

The key contributions of this paper are summarised as follows.

- The novel transmitter-receiver model has the ability to transmit an N number of subcarriers and receive the same amount. Additionally, our proposed system can also be used as SDR, where instead of using two antennas, one antenna can transmit and receive signals. Our system combines the advantages of both Wi-Fi and radar sensing. The power level, operating frequency, pulse repetition interval, antenna model, and radiation pattern can be changed at run-time. On the contrary, in Wi-Fi sensing, the radio frequency parameters are fixed, but the movement in the surroundings greatly affects their performance. Our system also presents useful amplitude and phase information, in comparison with Wi-Fi sensing, where only amplitude information can be used due to the presence of random noise. Self-designed antennas and off-the-shelf antennas (changed at the hardware) can also be used, depending on the application, making it flexible and scalable, both at software and hardware levels.
- The system is driven by a novel lightweight approach called extra-trees or extremely randomised trees (ERT). The ERT algorithm is an ensemble approach which not only reduces over-fitting and under-fitting issues but also the time complexity.

## Related Work

In recent literature, several contact and non-contact systems for respiration monitoring have been proposed. Wearable sensors and smartwatches, among other things, are required for a contact-based solution (Liaqat et al. 2019). Contact-based solutions can be costly and uncomfortable for some patients in terms of privacy and being attached to the body for long periods. In contrast to this, non-contact techniques based on radio-frequency (RF) sensing technology have recently been proposed, such as radar (Saeed et al. 2021c), Wi-Fi (Liu et al. 2018) and SDR (Shah et al. 2021). Continuous monitoring at home, even while sleeping, is one of the benefits of non-contact technology. Most non-contact solutions rely on vision (e.g., cameras) or RF (e.g., SDR) technology (Rehouma et al. 2019). The non-contact RF technology uses the way electromagnetic signals move through space and can be picked up over a wireless channel.

The radar, Wi-Fi, and SDR technologies are used in an RF-based respiration monitoring system (Saeed et al. 2022a). These radio-based systems can leverage channel state information (CSI) or received signal strength (RSS) for a variety of purposes, including detecting abnormal respiration. Furthermore, the FMCW radar and the Doppler radar are two techniques for the radar-based respiration monitoring system (Purnomo et al. 2021). These radar-based techniques need high-cost specialised gear capable of high-frequency operation. To detect breathing and heart rate, the vital-radio system uses an FMCW radar with a broad bandwidth of 5.46 to 7.25 GHz to track respiration and heart rate (Adib et al. 2015).

Several techniques employing RSS, CSI, and others for Wi-Fi-based respiration monitoring have been considered (Zhang et al. 2022, 2018). In Patwari et al. (2013), the authors investigated the measurement of RSS in radio device connections to determine an individual’s respiration rate and position in a home setting. Moreover, the authors of Abdelnasser et al. (2015) demonstrated a complete architecture to detect respiration signals from noisy Wi-Fi signals. Wi-Fi-based RF sensing provides various advantages, including low-cost and immediate availability. Nevertheless, it has some drawbacks, including flexibility and lack of scalability, as well as under-reporting of orthogonal frequency division multiplexing (OFDM) subcarriers (Shah and Fioranelli 2019).

Authors have experimented with an SDR-based respiration monitoring system (Rehman et al. 2021c). According to the different literature, SDR-based respiration monitoring is the most efficient of all RF-based respiration monitoring systems since it provides a scalable, portable, and flexible solution (Tuttlebee 2003). SDR technology also allows for the customisation of the transmit and receive power and operating frequency. Furthermore, signal processing and ML algorithms can be used efficiently for SDR-based systems. ML has been used by several authors to detect and monitor different respiration patterns (Lee et al. 2018). However, several studies were unable to achieve satisfactory precision and could only classify fundamental respiration patterns as normal and rapid (Saeed et al. 2022b). As a result, a reliable system for detecting and accurately identifying a wide range of respiration patterns is significant.

In our previous work (Yang et al. 2018a; Shah et al. 2018, 2019; Yang et al. 2018b, c), we have extensively worked on Wi-Fi sensing as well, specifically using the Wi-Fi phase sanitation algorithm to remove random noise. The reason for using this particular algorithm is due to the fact that Wi-Fi sensing phase information intrinsically carries extremely random noise and the phase sanitization algorithm needs to be used. On the contrary, SDR-based sensing provides useful amplitude and phase information for intricate biomechanical movements such as respiratory disorders. The Wi-Fi sensing also provides fixed subcarriers; 56 when Intel 5300 network interface card (NIC) is used and 64 in case of Athores NIC. The operating frequency of the Wi-Fi router (2.4 GHz and 5 GHz) is also fixed. Hence, the range resolution of Wi-Fi sensing is very low in comparison with SDR sensing due to the fixed operating frequency. On the contrary, the number of subcarriers that SDR used in our work is also flexible. The operating frequency of SDR is 70 MHz-6 GHz. We have used 256 subcarriers for respiratory pattern monitoring instead of the 64 provided by the Wi-Fi router. Wi-Fi sensing only offers the use of different antennas on the receiving side, while antennas on the transmitting side are usually fixed and cannot be changed. However, with SDR sensing, we have the liberty to use high-gain antennas on both sides for respiratory purposes in order to mitigate unwanted movements in the surroundings. Additionally, the data was collected recently, when we only had prior ethical approval to undertake measurements on healthy human subjects without underlying respiratory symptoms. Due to the recent COVID-19 situation, our ethical approval for patients with a respiratory disorder is still under review by the ethics committee. That is why we recruited healthy subjects and asked them to mimic different respiratory patterns that were successfully identified using SDR sensing in conjunction with the ensemble learning algorithm.

## Methodology

**Fig. 3:**
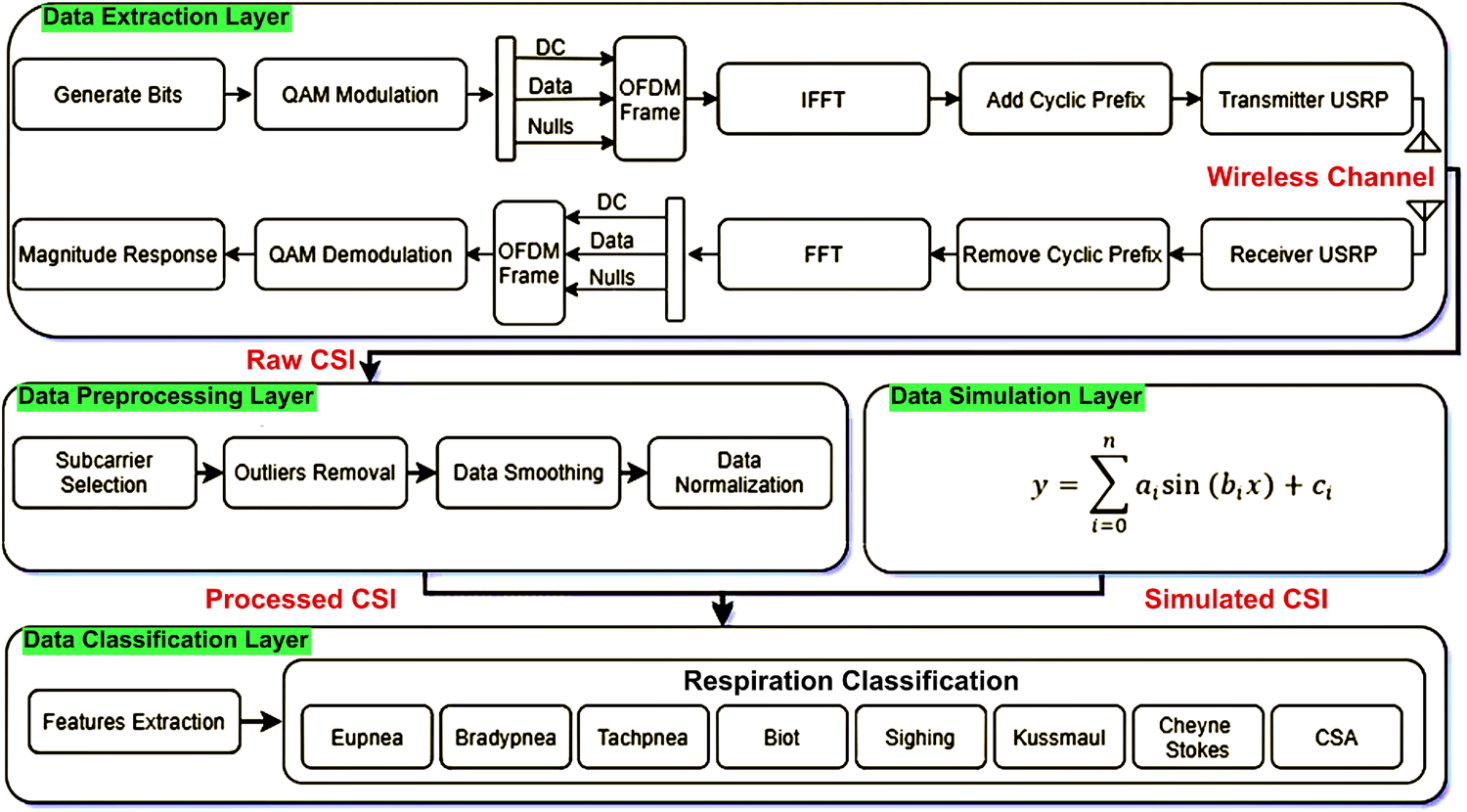
Proposed system architecture including different layers: data extraction, data preprocessing, simulation, and respiration classification

The data extraction, preprocessing, simulation, and classification layers are all depicted in Fig. 3, which outlines the proposed system architecture. Each layer is described in detail below.

### Extraction Layer

First, respiratory data are extracted. This layer has a transmitter, receiver, and radio channel. The transmitter includes a PC and a universal software radio peripheral (USRP) device. The transmitter PC assigns random bits to quadrature amplitude modulation (QAM) symbols. Each parallel frame receives data symbols. Reference symbols determine receiver channel estimate. One-by-one, border zeros are added. Inverse fast Fourier transform converts frequency domain to time domain. Repeating the final one-fourth of points in each frame creates a cyclic prefix (CP). CP eliminates offset of time and frequency.

Through gigabit ethernet, the host PC sends data to the USRP device. This information is digitally up-converted and then digital-to-analogue transformed. The USRP mixes an analogue signal with a user-set frequency using a low-pass filter (LPF). Before transmitting using an omnidirectional antenna, USRP equipment uses a transmit amplifier to manage signal intensity. The broadcast signal is picked up by an omnidirectional antenna at the receiving USRP device. A low-noise amplifier and a driving amplifier reduce noise and regulate gain. An LPF, a digital down converter and an analogue-to-digital converter are used in signal decimation. The signal is sent across gigabit Ethernet to the host PC. On the host PC, the Van de Beek method is used to remove CP, time, and frequency offsets from each frame (Beek et al. 1999). The fast Fourier transform is used to convert data from the time domain to the frequency domain. The amplitude response of frequency domain data is utilised to determine respiratory patterns.

As stated in Eq. 1, the wireless channel for the OFDM system can be considered as a narrow-band flat fading channel that can be described in the frequency domain.

1 $$\begin{aligned} \bar{Y}=H \times \bar{X}+\bar{N} \end{aligned}$$

where $\bar{X}$ and $\bar{Y}$ are the transmitted and received wireless signal vectors, respectively. $\bar{N}$ represents additive white Gaussian noise, and *H* represents the response of the OFDM channel frequency for all subcarriers calculated from $\bar{X}$ and $\bar{Y}$. On a 20-MHz channel, the OFDM system employs 256 subcarriers for data transmission. Equation 2 shows all subcarriers’ channel frequency response.

2 $$\begin{aligned} H=\left[ \begin{array}{cccc} H_{11} &{} H_{12} &{} \ldots &{} H_{1 s} \\ H_{21} &{} H_{22} &{} \ldots &{} H_{2 s} \\ \vdots &{} \vdots &{} \ldots &{} \vdots \\ H_{k 1} &{} H_{k 2} &{} \ldots &{} H_{k s} \end{array}\right] \end{aligned}$$

*k* and *s* are OFDM subcarriers and data samples, respectively. $H_{i}$ represents the channel’s frequency response for an individual subcarrier *i*. Equation 3 describes the complex value of it.

3 $$\begin{aligned} H_{i}=\left| H_{i}\right| \exp \left( j \angle H_{i}\right) \end{aligned}$$

$\left| H_{i}\right|$ and $\angle H_{i}$ represent the amplitude and phase responses of the OFDM subcarrier *i*, respectively. Equation 4 states the subcarrier *i* channel frequency response $H_{i}$ for indoor settings with multipath components.

4 $$\begin{aligned} H_{i}=\sum _{n=0}^{N} r_{n} \cdot e^{-j 2 \pi f_{i} \tau _{n}} \end{aligned}$$

where *N* is the total number of multipath components. The delays in attenuation and propagation in the $n_{th}$ path are indicated by $r_{n}$ and $\tau _{n}$, respectively. The frequency of the $i_{th}$ subcarrier is denoted by $f_{i}$.

### Preprocessing Layer

The data preprocessing layer receives unrefined CSI data from the extraction layer (Khan et al. 2021). This layer is subdivided further into four categories that are described as follows.

#### Subcarrier Selection

**Fig. 4:**
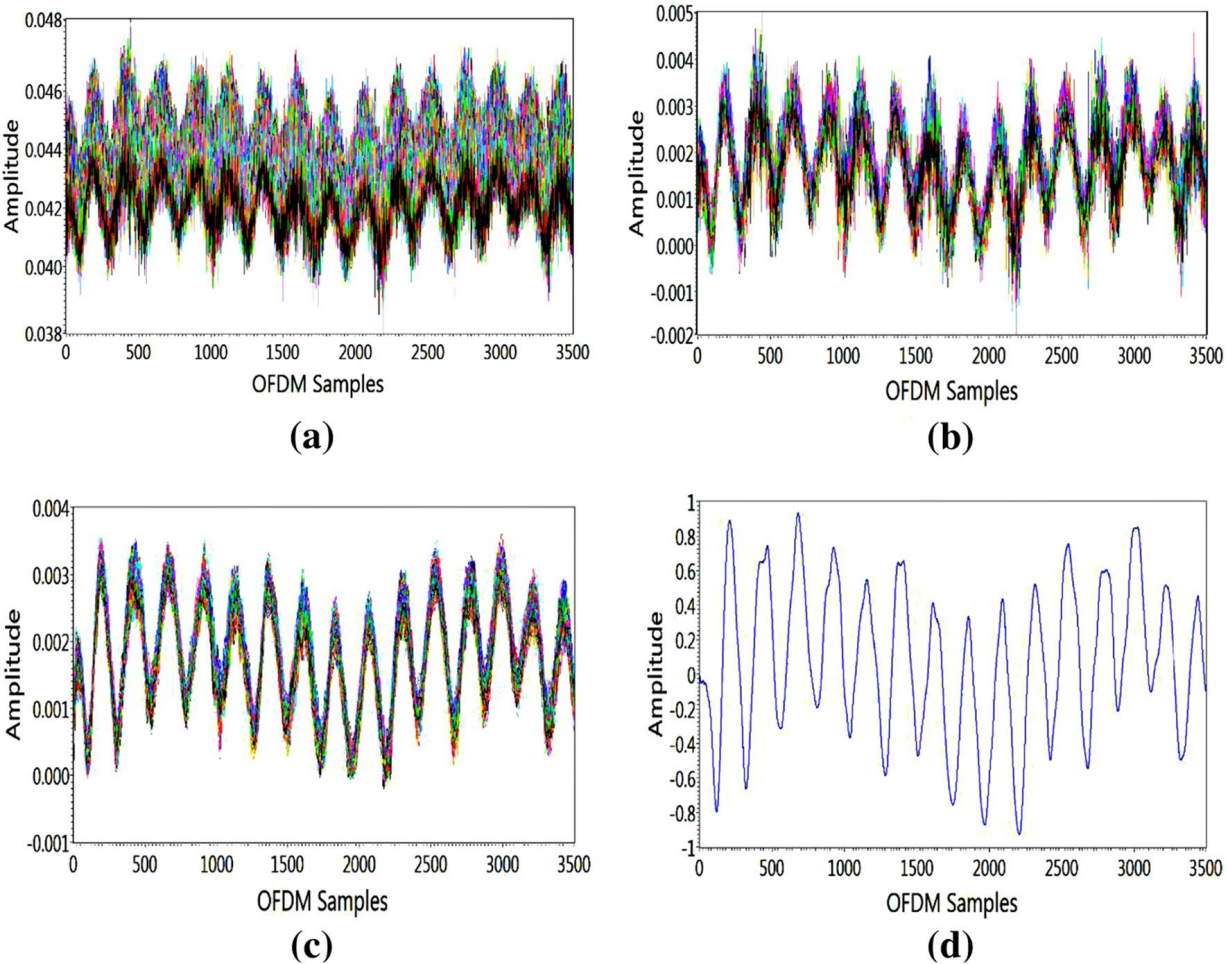
Signal preprocessing layers: **a** subcarrier selection **b** outliers expulsion **c** data refinement **d** data normalisation

The selection of subcarriers is the initial step in the data preprocessing layer. At the receiver, 256 OFDM subcarriers are captured after each respiration activity. For the respiration experiment, it is recognised that each subcarrier’s susceptibility is different. For accurate identification, all subcarriers with lower susceptibility to respiration activity must be eradicated, and therefore the variance of all subcarriers is calculated. As a result, less sensitive subcarriers are eliminated, as illustrated for all OFDM subcarriers in Fig. 4a.

#### Outliers Expulsion

The wavelet filtering is used after subcarrier selection to remove outliers from raw data while maintaining a crisp transition as illustrated in Fig. 4b. In coefficients, soft-heuristic SURE thresholding is used with a scaled noise option, syms5, and level four for wavelet filtering.

#### Data Refinement

For data refinement or smoothing, an eight-magnitude moving average filter is used. This reduces high-level frequency noise as illustrated in Fig. 4c. Equation 5 represents the result of the eight-magnitude window’s moving average filter.

5 $$\begin{aligned} y[n]=\frac{1}{N} \sum _{i=0}^{N-1} x[n-i] \end{aligned}$$

where *y*[*n*] represents the present output. The *x*[*n*] represents the current input, and *N* is the moving average filter’s window size.

#### Data Normalisation

Lastly, using Eq. 6, the waveform data is normalised to the highest and lowest values of 1 and $-1$, respectively.

6 $$\begin{aligned} \overline{y[n]}=\frac{y[n]-o f f s e t}{s c a l e} \end{aligned}$$

where *y*[*n*] are the input data and $\overline{y[n]}$ is the normalised data. To obtain a normalised waveform, the input waveform is offset and scaled by specified values. Figure 4d illustrates the normalised waveform for an individual OFDM subcarrier. After completing the aforementioned steps, the processed CSI data is used to identify distinct respiration patterns using a supervised ML classification algorithm.

### Simulation Layer

A reliable ML model requires several real-time respiration experiments. A simulation model has been constructed to address the data scarcity. A simulated model is built using authentic real-time respiration pattern data to provide high-quality simulated respiration data. Inhaling and exhaling are a continuous process, hence wireless respiration signals can be computed using sinusoidal waveforms. In this case, MATLAB’s curve fitting tool fits breathing patterns. Curve fitting uses a function to describe experimental data and derive model parameters. Equation 7 describes real-time respiration patterns using sinusoidal terms.

7 $$\begin{aligned} y=\sum _{i=0}^{n} a_{i} \sin \left( b_{i} x+c_{i}\right) \end{aligned}$$

**Fig. 5:**
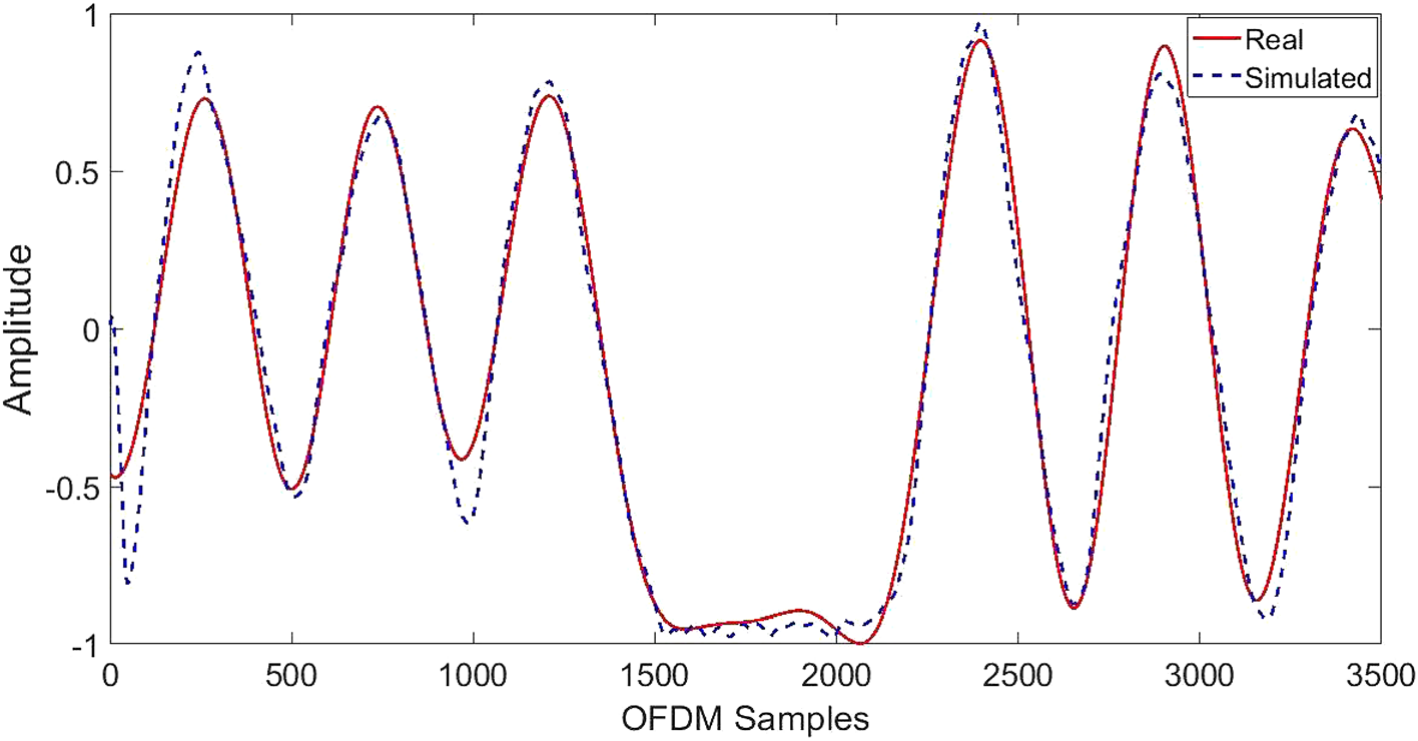
Sample of actual versus simulated Biot respiration pattern

For each sinusoidal term, *a* denotes the amplitude, *b* the frequency, and *c* the phase. Whereas *n* is the sinusoidal terms total number, and *x* represents OFDM samples. For demonstrative purposes, Fig. 5 depicts the actual and simulated Biot respiration patterns in real-time. Real-time breathing patterns are susceptible to oscillations. To precisely reproduce real-time data, the noise function of additive white Gaussian is utilised to incorporate noise into simulated respiration patterns. Slight changes in the parameters of the additive white Gaussian noise function can result in a large amount of simulated data. Simulated CSI for eight respiration patterns is created at the data simulation layer’s output and used for ML classification.

### Classification Layer

The simulated and processed CSI data combined is used for training, validating, and testing in this data classification layer. The outcome of the ML algorithm is assessed based on the following evaluation metrics: accuracy, F1-score, training time, and confusion matrix (Hossin and Sulaiman 2015). In this study, the number of data sets is increased by adding a large amount of simulated data to test how well the ML algorithm works.

## Experimental Results and Discussion

### Experimental Setup

**Fig. 6:**
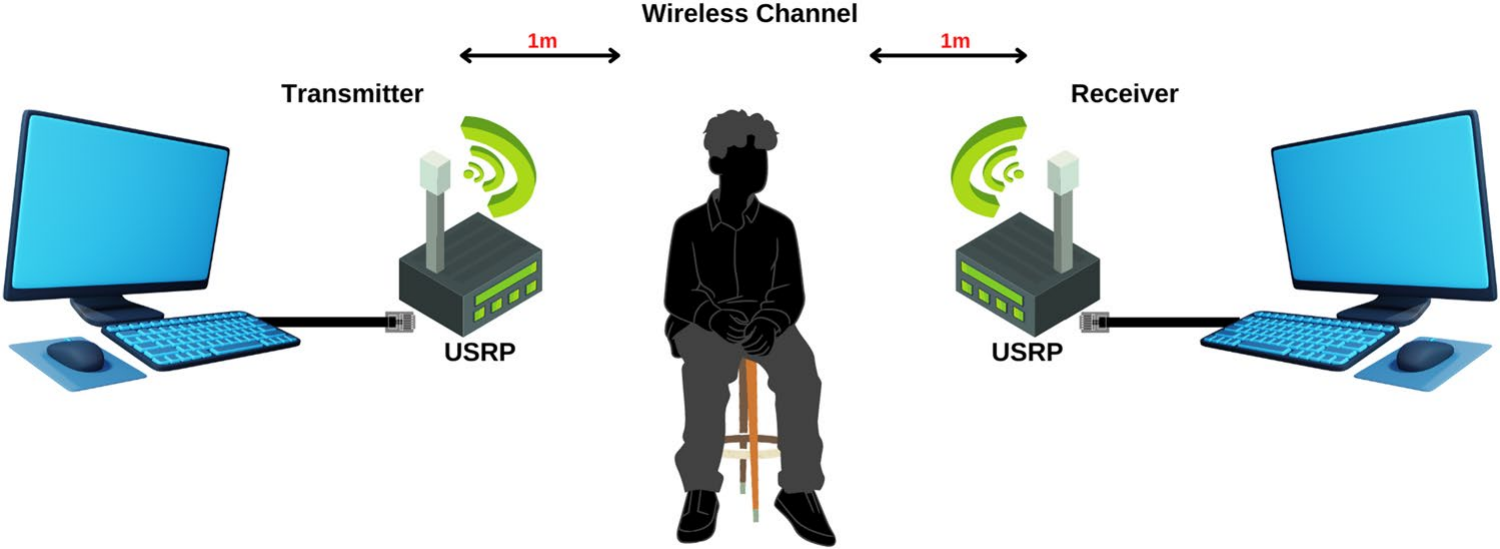
Wireless respiration monitoring setup based on SDR sensing

The experimental and data collection setup is shown in Fig. 6. The device detects chest oscillations to differentiate eight breathing patterns in real-time using fine-grained CSI. The transmitter, receiver, and wireless channel are the primary components of the experiment. PC and USRP function as transmitter and receiver. Omnidirectional antennas detect variations in CSI produced by breathing. In the laboratory setting, the transmitter’s RF signal traverses multiple ways to reach the receiver. Human bodies deflect RF signals, providing an additional path. On the receiver side, human breathing influences signal propagation as CSI.

**Table 1:** Volunteers detail who participated in the experimental study

No.	Gender	Age	Weight (kg)	Height (cm)	Body Mass Index (BMI)
1	Male	26	76	172	25.4
2	Male	28	65	180	20.3
3	Male	31	51	177	16.8
4	Male	31	51	175	16.6
5	Male	31	64	172	21.5

Tab. 1 contains information about five healthy individuals who participated in this research and performed varied human respiratory patterns. As seen in Fig. 6, each participant was seated in a static position while collecting data. Transmitter and receiver USRPs are at 100 cm from the volunteer’s abdomen. Each breathing pattern has extensive guidelines for volunteers to get accurate data. Each participant’s respiratory activity is recorded for 30 s.

### Respiration Identification

**Fig. 7:**
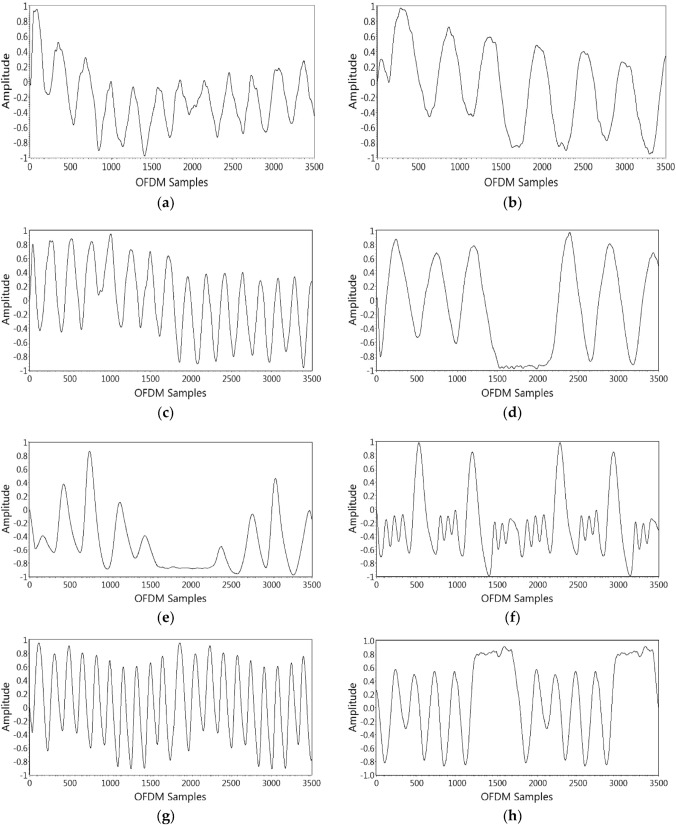
Respiration patterns outcome: **a** eupnea, **b** bradypnea, **c** tachypnea, **d** biot, **e** sighing, **f** cheyne-stokes, **g** kussmaul, **h** central sleep apnea (CSA)

This paragraph describes how to use SDR-based RF sensing to monitor respiration patterns. The respiration patterns are analysed using the CSI amplitude response. For each respiration experiment, the fluctuations in the frequency response of the amplitude are measured in 3500 OFDM samples. For demonstration purposes, one of the volunteer’s results for eight respiration patterns is shown in Fig. 7 for an individual OFDM subcarrier. Regular respiration at a consistent pace and pattern of 10–24 breaths per minute is defined as eupnea. Figure 7a shows that a half-minute has 12 breaths that are within the normal respiration range. Bradypnea is defined as a shallow and slow respiratory pattern with a consistent pattern. Figure 7b shows 6 breaths in a half minute, which is considered sluggish respiration. The respiration rate is quicker in tachypnea than in eupnea, as can be seen in Fig. 7c, when 15 breaths are taken in a half minute. Biot is characterised by deep respiration followed by apnea regularly, as can be seen in Fig. 7d. Sighing is simply normal respiration with pauses for sighs. Figure 7e illustrates this with regular respiration interrupted by periodic deep breaths. Cheyne-stokes is characterised by a progressive increase and reduction in the respiration rate, as can be seen in Fig. 7f. Figure 7g illustrates Kussmaul respiration that is both deep and rapid. In addition, CSA is a type of respiration that stops and resumes periodically throughout sleep, as can be seen in Fig. 7h, which depicts intervals of no breath during regular respiration.

**Fig. 8:**
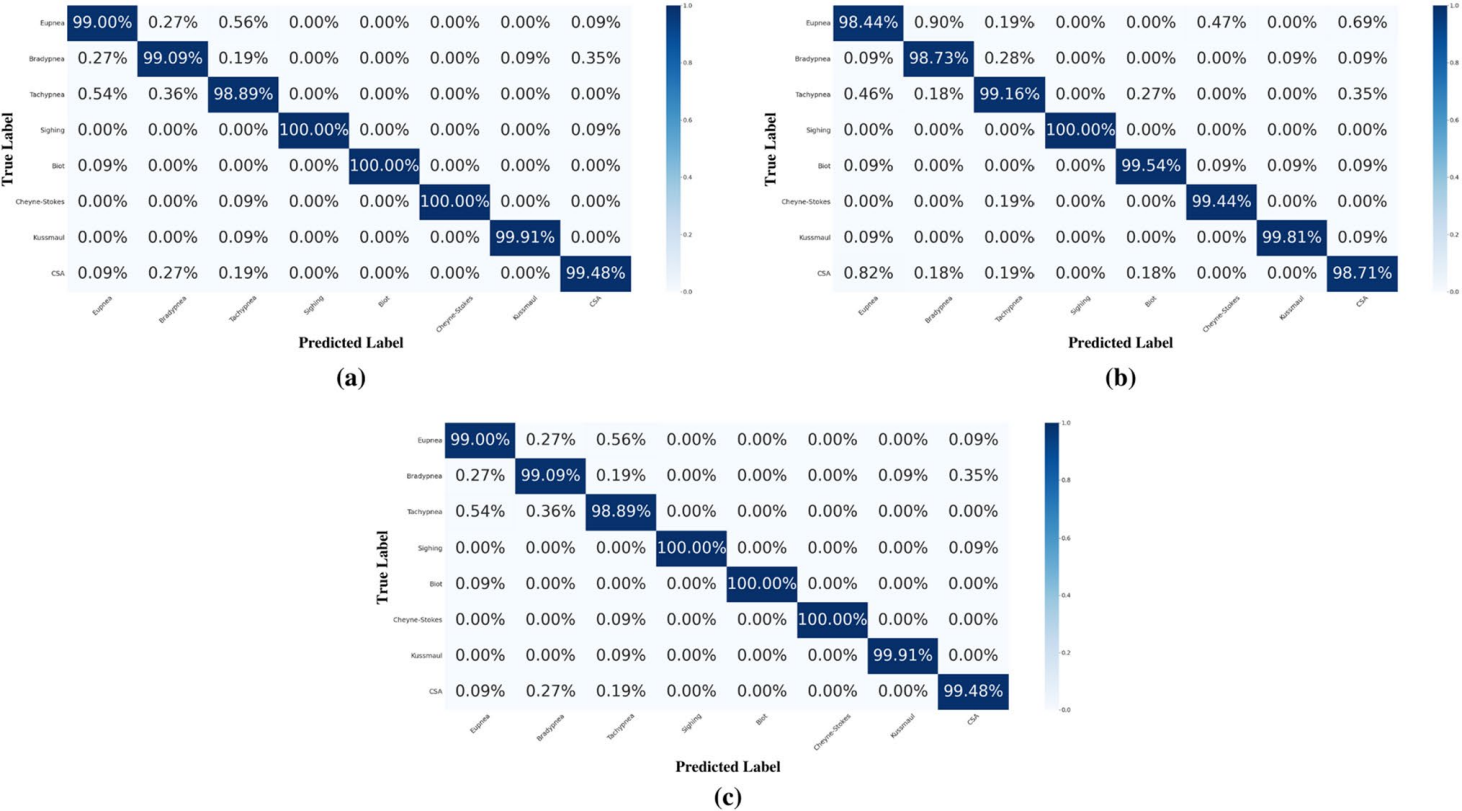
Respiration classification results in the form of a confusion matrix: **a** extremely randomised trees, **b** decision tree, and **c** multilayer perceptron

This paragraph discusses the findings of ML algorithms for eight different human respiration datasets. Performance evaluation metrics include accuracy, F1-score, training time, and confusion matrix. To design a lightweight real-time system, the training time of an algorithm plays a key role. In this study, an ensemble learning-based technique ERT (Geurts et al. 2006) is investigated along with decision tree (DT) and multilayer perceptron (MLP). These algorithms (or classifiers) are trained using the Python programming language, primarily through the scikit-learn and TensorFlow libraries. From the final dataset of the acquired respiration data, 70% is used to train an algorithm and 30% to validate and test it. Figure 8 presents the findings of the confusion matrix that reveal that ERT, DT, and MLP precisely identified eight different respiration classes. The diagonal values of the confusion matrix reflect events in which the actual and predicted classes are matched. Other values reveal the misclassification error of the trained ML model. Furthermore, Tab. 2 presents the findings in terms of accuracy, F1 score, and training time. As can be seen, all classifiers achieved high accuracy and an F1 score of up to 99%. Nevertheless, ERT revealed the lowest training time of only 0.71 s compared to DT (82.25 s) and MLP (163.94 s). This outcome demonstrates the effectiveness of an ensemble learning technique, such as ERT, to design a lightweight real-time system for the identification of anomalous respiration.

**Table 2:** Machine learning-based findings for eight diverse human respiration

Classifier	Accuracy	F1-score	Training time
Extremely randomised trees	$\approx$ 99%	$\approx$ 99%	0.71 s
Decision tree	$\approx$ 98%	$\approx$ 98%	82.25 s
Multilayer perceptron	$\approx$ 99%	$\approx$ 99%	163.94 s

## Conclusion

In this paper, a lightweight real-time solution is proposed for the identification of anomalous respiration using the USRP wireless sensing device and the ERT ensemble learning technique. Additionally, a solution to the challenge of gathering respiration data is proposed. Based on CSI variations, a wireless SDR platform is used to capture real-time human respiration data. Using the curve fitting approach, the acquired real-time respiration data is used to produce large amounts of simulated data. The simulated data, along with the actual acquired data, improved the performance of the ML model. The ML techniques (ERT, DT, and MLP) are used to identify eight different respiration patterns. The experimental findings reveal that the proposed platform is accurate, lightweight, and reliable in a static setting to monitor human respiration. The proposed platform’s potential applications include monitoring respiratory conditions in a case of chronic obstructive pulmonary disease, asthma, coronavirus, and possibly future pandemics.
